# Recent advancements in multifaceted roles of flavonoids in plant–rhizomicrobiome interactions

**DOI:** 10.3389/fpls.2023.1297706

**Published:** 2024-01-05

**Authors:** Gokul Anil Kumar, Sumit Kumar, Rupesh Bhardwaj, Prashant Swapnil, Mukesh Meena, Chandra Shekhar Seth, Ankush Yadav

**Affiliations:** ^1^ School of Basic Science, Department of Botany, Central University of Punjab, Bhatinda, Punjab, India; ^2^ Department of Mycology and Plant Pathology, Institute of Agricultural Sciences, Banaras Hindu University, Varanasi, Uttar Pradesh, India; ^3^ Department of Plant Pathology, B.M. College of Agriculture, Khandwa, Rajmata Vijayaraje Scindia Krishi Vishwa Vidyalaya, Gwalior, India; ^4^ Laboratory of Phytopathology and Microbial Biotechnology, Department of Botany, Mohanlal Sukhadia University, Udaipur, Rajasthan, India; ^5^ Department of Botany, University of Delhi, New Delhi, Delhi, India

**Keywords:** mycorrhiza, plant growth promoting rhizobacteria (PGPR), *Rhizobium*, rhizosphere, secondary metabolites, stress

## Abstract

The rhizosphere consists of a plethora of microbes, interacting with each other as well as with the plants present in proximity. The root exudates consist of a variety of secondary metabolites such as strigolactones and other phenolic compounds such as coumarin that helps in facilitating communication and forming associations with beneficial microbes in the rhizosphere. Among different secondary metabolites flavonoids (natural polyphenolic compounds) continuously increasing attention in scientific fields for showing several slews of biological activities. Flavonoids possess a benzo-γ-pyrone skeleton and several classes of flavonoids have been reported on the basis of their basic structure such as flavanones, flavonols, anthocyanins, etc. The mutualistic association between plant growth-promoting rhizobacteria (PGPR) and plants have been reported to help the host plants in surviving various biotic and abiotic stresses such as low nitrogen and phosphorus, drought and salinity stress, pathogen attack, and herbivory. This review sheds light upon one such component of root exudate known as flavonoids, which is well known for nodulation in legume plants. Apart from the well-known role in inducing nodulation in legumes, this group of compounds has anti-microbial and antifungal properties helping in establishing defensive mechanisms and playing a major role in forming mycorrhizal associations for the enhanced acquisition of nutrients such as iron and phosphorus. Further, this review highlights the role of flavonoids in plants for recruiting non-mutualistic microbes under stress and other important aspects regarding recent findings on the functions of this secondary metabolite in guiding the plant-microbe interaction and how organic matter affects its functionality in soil.

## 1 Introduction

Flavonoids are one of the most prominent classes of polyphenolic compounds synthesized by the various biosynthetic pathways such as shikimic acid pathway, phenylpropanoid pathway and flavonoids pathways (Cesco et al., 2012). The synthesis of phenylalanine by shikimic acid pathway undergoes phenylpropanoid pathway and synthesize 4-coumaroyl-CoA. Through condensation reaction and with the help of chalcone-synthase 2′,4′,6′,4-tetrahydroxy chalcone synthesis takes place. Due to isomerization by the action of an isomerase (chalcone-flavanone), 2′,4′,6′,4-tetrahydroxy chalcone form flavanone which initiates the flavonoid pathway to produce the several classes of flavonoids (Cesco et al., 2012; Liga, et al., 2023). This class consists of a variety of members including phytoalexins, flavonols, flavones, flavanones, isoflavonoids, anthocyanins, anthoxanthins, chalcones and proanthocyanidins (Liga et al., 2023).

**Figure 1:**
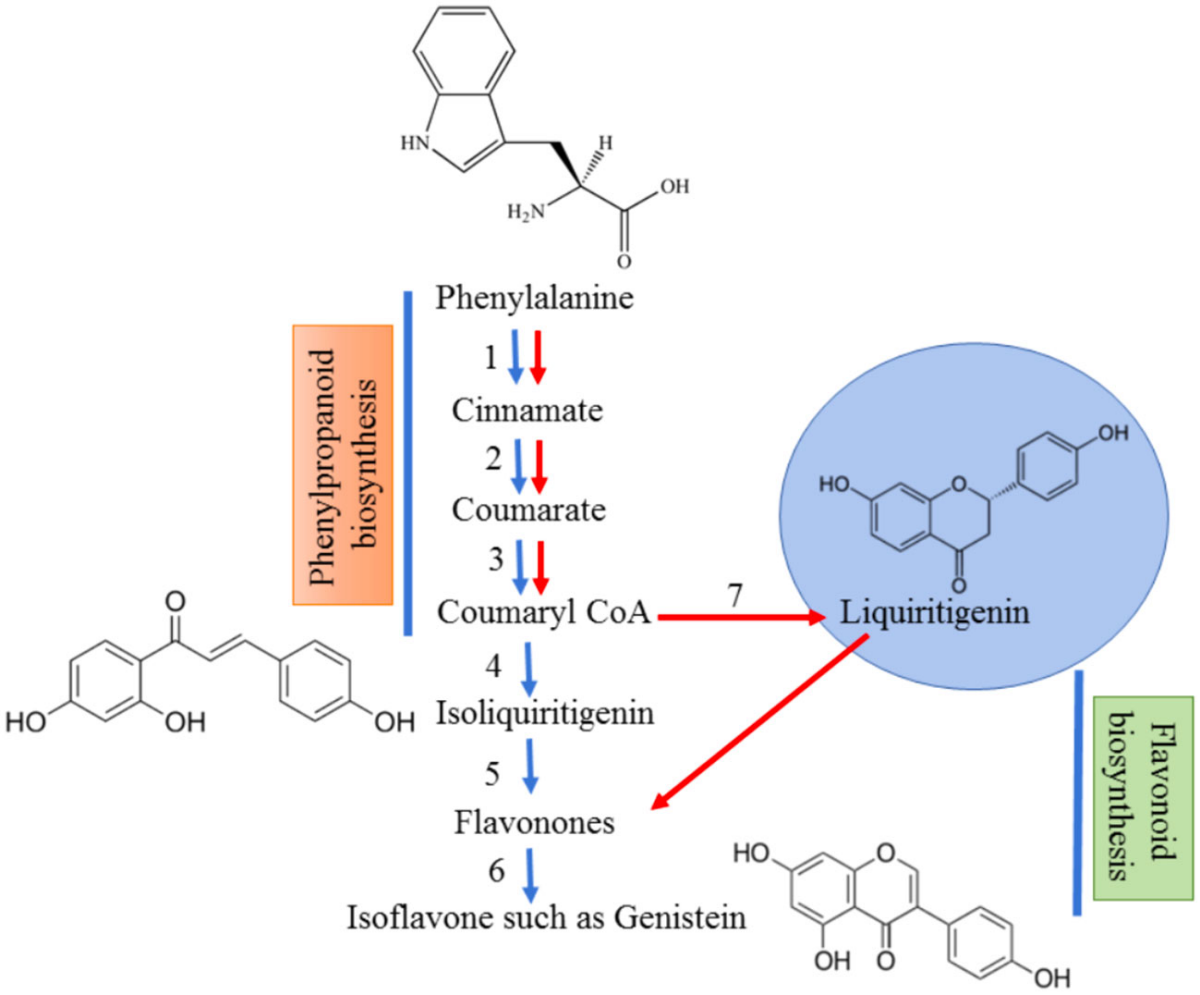
Biosynthesis of isoflavonoids from precursor, phenylalanine. The red arrows designate the normal pathway for the production of flavonoids whereas the blue arrows show the pathway for isoflavonoid synthesis. Phenylalanine is converted to coumaryl CoA by the help of 3 enzymes – (1) Phenylalanine ammonia lyase; (2) Cinnamate 4 hydroxylyase; (3) 4-coumarate CoA ligase. Coumaryl CoA gets converted to isoliquiritigenin which is mediated by a novel enzyme known as chalcone reductase (4) in combination with chalcone synthase (4,7). The flavones are formed by the action of chalcone isomerase (5) which is converted to isoflavones by isoflavone synthase (6).

**Figure 2:**
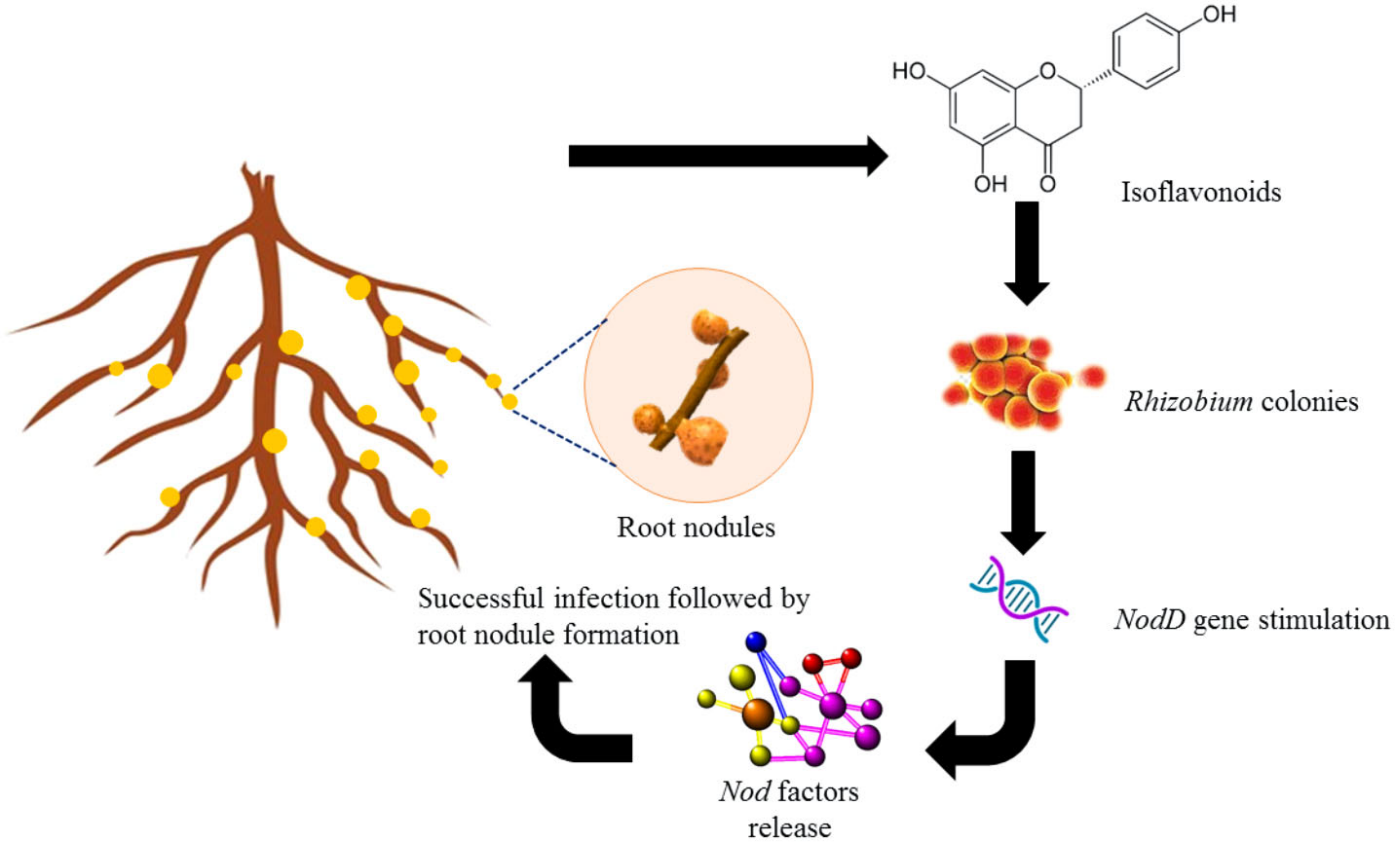
Role of isoflavonoids such as naringenin and genistein in inducing nodulation in roots of legumes.

The biosynthesis of flavonoids involves enzymes chalcone synthase (CHS) and chalcone isomerase (CHI) and a specific type of chalcone isomerase that is found in legumes (Liu and Murray, 2016). This leads to the formation of isoflavonoids, which is a distinctive compound of leguminous plants (**Figure 1**). Flavonoids can be secreted in two ways into the rhizosphere, an active way involving ABC transporter utilizing adenosine triphosphate (ATP) and a passive way which includes degradation of root cap and cortical cells (Shah and Smith, 2020). Apart from acting as UV filters for terrestrial plants, the principal function of flavonoids identified in legumes is inducing nodule formation in the roots by triggering the *nodD* genes in the bacteria (Chandran et al., 2021; Nascimento and Tattini, 2022) which further activates other *nod* genes to establish mutualistic relations with *Rhizobia* (**Figure 2**). Though a wide variety of molecules have been recognized within this class, very few have shown potential to induce nodulation since specific type of (iso)flavonoid is secreted by roots which gives this interaction a host-specific nature (Rosier et al., 2016).

The case of flavone luteolin and chalcone 4,4′-dihydroxy-2′-methoxychalcone (methoxychalcone) in the *Medicago sativa* L.*-Sinorhizobium melitoli* symbiosis is noteworthy here (Liu and Murray, 2016). Luteolin is not legume-specific and is found in many plant families. Though, the external application of luteolin to this symbiosis enhances the nodulation process by triggering *nod* genes, the flavonoid is not specific to the *M. sativa-S. melitoli* symbiosis. However, methoxychalcone is one of the few isoflavonoids found in root exudates which is produced from isoliquiritigenin by the enzyme chalcone *O*-methyl transferase (ChOMT) and induces the *nod* genes (Liu and Murray, 2016). Apart from their classical function of inducing nodulation, they are also known to play an additional role in phosphorus and iron acquisition as well as in auxin biosynthesis and blocking auxin transporters causing nodule formation by localization of auxin as a result (Gifford et al., 2018). They are also known for facilitating pollination, UV protection and possess antioxidant properties.

Plants are often found in association with a diverse array of microorganisms inhabiting different parts of plants and in soil – a region known as rhizosphere. This microbiome can be found associated with three different types of niches such as phyllosphere, endosphere, and rhizosphere (Rosier et al., 2016). The plant-microbe interactions have mostly been focused on rhizospheric microflora than the other two niches on account of the complex interactions between bacteria and plants including the prospective positive effects on plant health and growth (Kumari et al., 2018; Chandran et al., 2021; Kumar et al., 2022). The roots of the plants secrete root exudates, containing various chemical signals, one of which is our compound of interest, flavonoids. The bacterial population responds to this secretion which acts as a signaling molecules as well as source of nutrients for them (Ma et al., 2016; Seth et al., 2023). It has been seen that though a large number of flavonoids are produced in a plant, the compounds have to be secreted from the roots into the rhizosphere and they are very specific in function (Liu and Murray, 2016). This attracts a particular bacterial population to the host plant, which encourages them to colonize the plant (Santoyo et al., 2021; Mori et al., 2023).

It has been found that such associations are formed under biotic and abiotic stresses such as low N_2_ levels in soil leading to selective attraction of *Rhizobium* to the legumes but the genetic contribution of the production of specific flavonoids are still in progress (Rosier et al., 2016). It has been deduced that the amount of secretion of flavonoids secreted into the rhizosphere has a direct correlation with the amount of nutrients in the soil and helps in avoiding energy expenditure in unnecessary symbiotic association (Cesco et al., 2012). Studies have found that apigenin and luteolin act as chemical signals when the plant experiences nitrogen deficiency for attracting *Rhizobia* for nodulation (Abedini et al., 2021). Within the soil, the bacteria-bacteria interaction is prevalent and takes place by a phenomenon called quorum sensing (QS) and it involves communication between two bacterial individuals by certain compounds known as auto-inducers such as *N*-acyl-*L*-homoserine lactones (AHL) (Ma et al., 2016; Zehra et al., 2021).

Plants can also sense the presence of AHLs in the soil and secrete exudates in response to these signals which can inhibit or promote the diversity of microbes. This process is known as quorum quenching and is achieved by molecules that imitate AHL or enzymes that destroy AHLs. It is rather unclear whether the flavonoids are responsible for quorum quenching or not. However, certain reports suggest that they promote QS which further accelerates the nodulation process, the importance of which has been highlighted by the findings in *Sinorhizobium fredii* (Pérez-Montaño et al., 2011). Flavanones such as naringenin decrease the production of QS molecules helping in decreasing the virulence of *Pseudomonas aeruginosa* (PA01), a pathogenic microbe (Vandeputte et al., 2011). Thus, it can be concluded that flavonoids can selectively regulate the virulence of beneficial and pathogenic bacteria. Also, it has been speculated that rhizobial infection further modifies the overall flavonoid profile of the host by promoting the synthesis of favorable compounds such as genistein and daidzein that protects the *Rhizobia* that can help in infection and protects them from the production of phytoalexins (Cesco et al., 2010). In response to a successful plant-microbe interaction which is possibly speculated to act as a selective response to other potential infection agents as seen in case of *S*. *melitoli* which is resistant to medicarpin unlike *Bradyrhizobium* and other heterologous forms (Liu and Murray, 2016).

The most significant function of flavonoids, particularly isoflavonoids, is root nodulation which forms the foundation for a symbiotic association. It has been found that flavonoids have to be released from the roots into the rhizosphere to perform this function and have a tendency to play a pivotal role in host-microbe specificity (Weston and Mathesius, 2013). Though bacteria-bacteria communication happens by a phenomenon called QS, it is still an enigma whether flavonoid is a quorum quencher or stimulator. Reports mentioned in this review suggest that compounds such as naringenin help in the selective attraction of microorganisms. Flavonoids have been famous for their role in inducing nodulation in legume plants (Bosse et al., 2021). Though a lot has been investigated about N_2_ fixation, the genetic network of regulation of secreting flavonoids in attracting *Rhizobia* is still unexplored and still needs to be studied.

Apart from this, these compounds facilitate the formation of mycorrhizal associations. Though there has been doubt regarding the degree of involvement of these molecules in attracting the fungal mycelium toward roots, the extent of their participation needs to be worked upon. There has been a bone of contention between two schools of researchers that whether these metabolites are necessary for arbuscular mycorrhiza (AM) fungal colonization or not as the evidences reported till now are unable to give a clear picture. An elaborate study on the necessity of flavonoids in AM fungal colonization would help in protecting the plants from severe stress conditions by exogenous application of the same. Associations with other plant growth promoting rhizobacteria (PGPR) have also been studied and results have been quite promising. The concentration of released flavonoids as well as the nature of the compounds determine the formation of this association. These methods help with the acquisition of nitrogen and phosphorus. They also have an additional role in iron acquisition by acting as a chelating agent. After the release, the flavonoids are degraded by biotic and abiotic means. The glycosidic derivatives of flavonoids are degraded by the enzymatic hydrolysis of glycoside by the microorganisms, rendering it impotent. The abiotic factors such as organic matter content in the soil have been recently found to contribute to the inhibition of the molecular dialogue (Qiu et al., 2018). Substantial research needs to be done to explore the potential abiotic factors that affect bioavailability. The ‘Cry for help’ hypothesis advocated by some researchers has had a significant impact in understanding the immune responses of the plants to an immediate crisis (Rolfe et al., 2019; He et al., 2022). However, the extent of the usage of this distress call is unknown since in some cases flavonoids have been reported to only enhance the pre-established mutualism between two parties rather than recruit a potential ally. Therefore, the validity of the above-mentioned hypothesis needs to have a set limit. Most importantly, the availability and persistence of flavonoids in the soil need to have substantial proof from the abiotic factor point of view since very limited information is available on the effect of soil parameters on the life of this compound.

This review paper digs into the recent findings of the roles and functions of this ubiquitous compound in plant-microbe interactions and the effects of abiotic and biotic stresses on its functioning.

## 2 Classification of flavonoids

**Figure 3:**
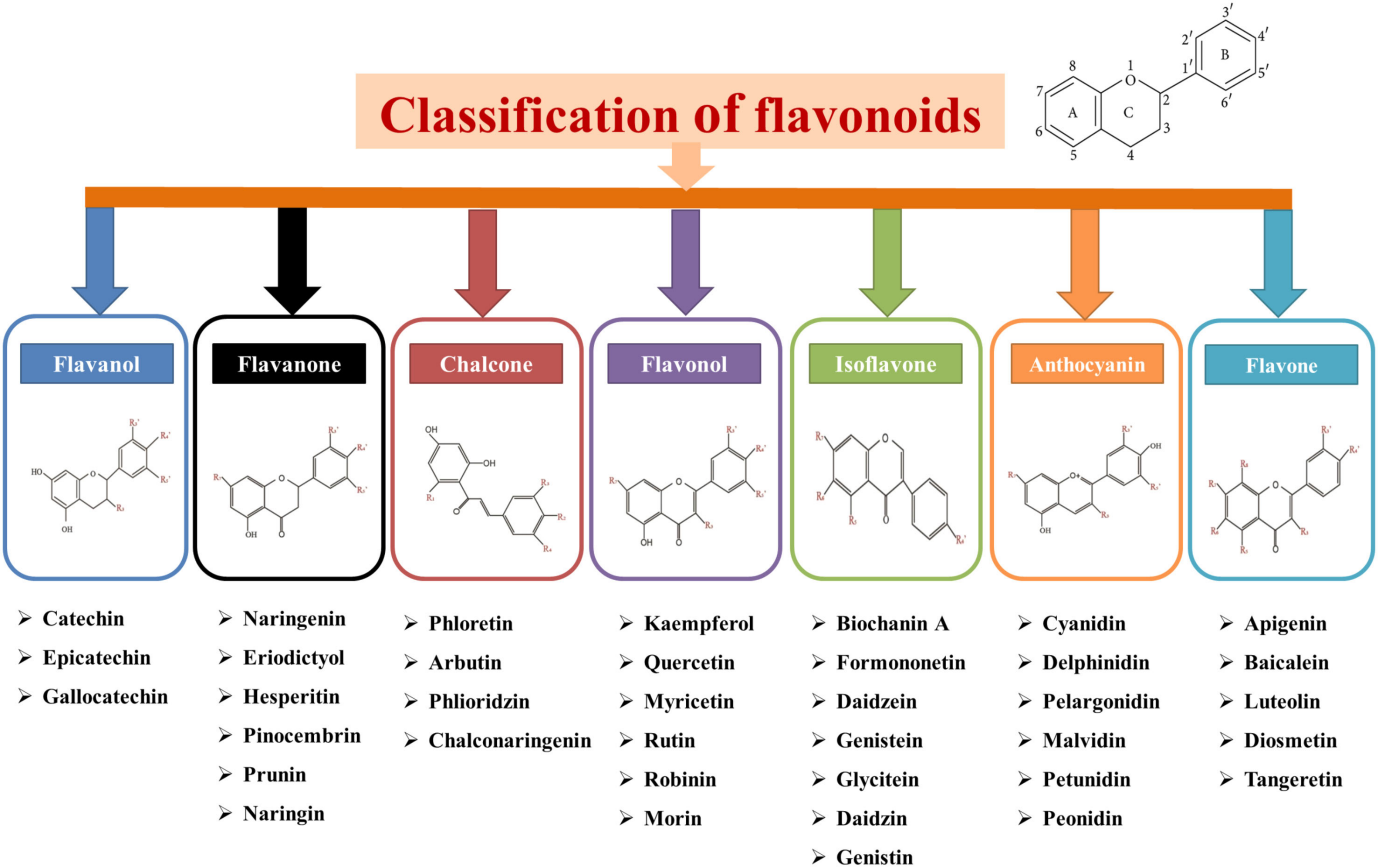
Basic structure and classification of flavonoids with chemical structure and examples.

Flavonoids are included in a diverse group of low-molecular-weight phenolic compounds or polyphenols that are mainly found in the ubiquitous plant kingdom (Samanta et al., 2011; Roy et al., 2022). The chemical structure of flavonoids are usually made up of 15 carbon atoms, which form their basic skeleton. Basically, all flavonoids are demonstrated by a C6-C3-C6 chemical structure comprising two benzene rings, A and B, connected by a heterocycle-pyrene ring (C) that contains oxygen (Falcone Ferreyra et al., 2012; Mierziak et al., 2014). The basic structure of flavonoids are shown in **Figure 3**. Further, flavonoids can be divided into seven different subclasses based on their structural differences, which are presented in **Figure 3**. This classification is based on the pattern of their central heterocyclic pyran ring in their core flavan structure.

Furthermore, in plants, flavonoids typically assemble in the free form (aglycones) or are linked to sugars, but their derivatives are mainly synthesized by different processes such as glycosidation, methylation, and polymerization, which directly or indirectly affects their performance (Wen et al., 2017; Yang et al., 2018). The most common type of flavonoid derivatives are glycosides; the least found are *O*-glycosides; and *C*-glycosides are the rarest form (Rauter et al., 2007). These glycosylated flavonoids are the most significant; for instance, glycosylated anthocyanidins are acknowledged as an essential class of flavonoids, anthocyanins (Smeriglio et al., 2016). In actuality, anthocyanidins are found bound to sugars and are light-sensitive by aiding transport through the membrane, glycosylation improves solubility, biodistribution, and metabolism, and methylation boosts the entry of flavonoids into the cells and protects them (Šamec et al., 2021). In general, it has been reported that methylated flavonoids are less abundant compared to free-form flavonoids or flavonoid glycosides (Wen et al., 2017). The two most common methylation patterns of flavonoids are *C*-methylation and *O*-methylation (Chandran et al., 2022).

## 3 Mechanisms of flavonoids secretion into the rhizosphere

**Figure 4:**
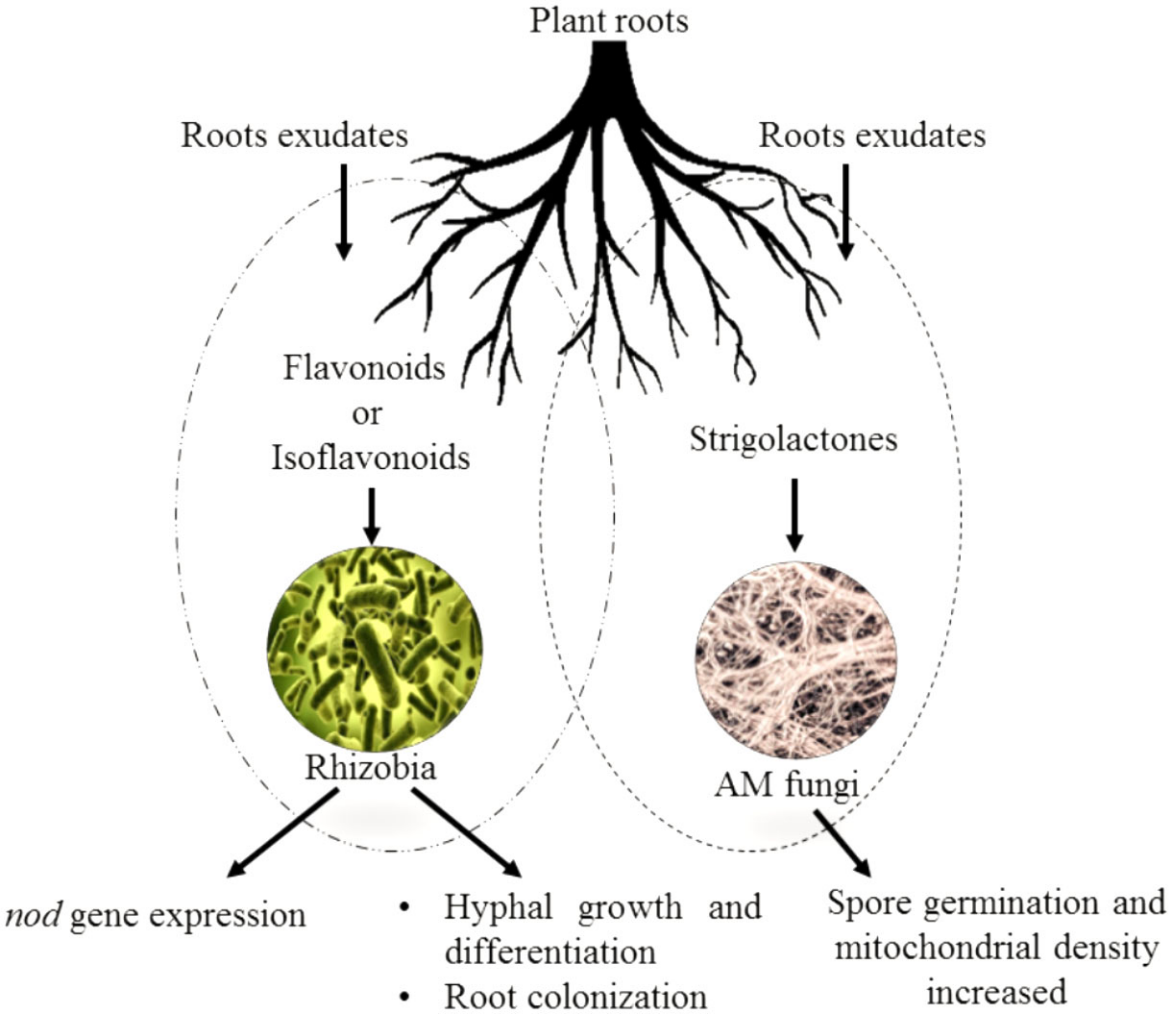
Effect of flavonoids and isoflavonoids on establishment of symbiotic associations such as plant/*Rhizobium* and plant/AMF. Isoflavonoids in the former association help in *nod* gene expression releasing nod factors forming nodules and successful infection in host roots. The flavonoids in latter helping attracting the germinating hyphae to the roots and cause further branching and differentiation.

Flavonoids are a group of phenylpropanoid metabolites synthesized *via* the *p*-coumaroyl-CoA and malonyl-CoA pathways (Ferrer et al., 2008). In plants, almost all flavonoids accumulate in vacuoles as glycosides, whereas some are released by the roots into the rhizospheres. Released flavonoids in the rhizosphere can play multifunctional roles, such as protecting plants from biotic and abiotic threats (Wang et al., 2022). It is reported that some important transporter families are responsible for the vacuolar accumulation of glycosides, for instance, vesicle-mediated transporters or membrane-bound transporters of the ABC (ATP-binding cassette) and multidrug and toxic compound extrusion (MATE) families (Shoji, 2014). It has been proposed that ABC transporters facilitate the release of flavonoids into the rhizosphere. Flavonoids are actively exuded from roots in response to various elicitors. In general, two important transport mechanisms play polar roles in flavonoid secretion from roots (Bertin et al., 2003; Shaw et al., 2006). First is the active transport mechanism, which is root exudation; and second is the passive transport mechanism, which is root turnover, root injury, and root decomposition from root cap and border cells (**Figure 4**).

Plasma membrane vesicles are produced from soybean roots, and these roots possess an ATP-dependent transporter that facilitates in the secretion of genistein into the rhizosphere (Sugiyama et al., 2007). The major candidate for this pathway, according to biochemical transport experiments utilizing different inhibitors, is an ABC transporter. This ABC-type transporter is most specific for isoflavone aglycones, but it does not transport isoflavone glucosides. According to these findings, aglycones and glucosides are secreted from soybean roots through several processes. In the cytosol of soybean, three different isoflavone aglycone types—daidzein, genistein, and glycitein—are produced and their glucosides and malonylglucosides are likely collected in the vacuoles. These aglycones are also secreted from roots and found in root exudates (Sugiyama, 2019). In soybean roots, it has been discovered that apoplastic β-glucosidases release isoflavones from their conjugates. This may be a crucial mechanism for the release of active flavonoid aglycones during root-microbe interactions (Suzuki et al., 2006). The efflux of isoliquiritigenin from tobacco BY-2 cells expressing MtABCG10 was observed (Biała et al., 2017).

Aglycones are a more active form of isoflavonoids that help induce legume-rhizobial interactions and activate defense systems against phytopathogens in the rhizosphere. Apoplastic β-glucosidase may assist plants in quickly secreting significant amounts of the active flavonoids, which are stored in vacuoles, into rhizospheres. By inducing the expression of *nod* genes in rhizobial bacteria, isoflavones released by legume roots into the rhizosphere trigger a cascade of root-bacteria symbiotic interactions that result in infection and the curling of root hairs, the production of infection threads, and the development of nodules in root cortical cells near to the vascular tissues (Subramanian et al., 2006; Hassan and Mathesius, 2012). For instance, isoflavones like daidzein and genistein are capable of inducing *nod* genes in the *Rhizobia* and facilitating symbiosis for biological nitrogen fixation (Pueppke et al., 1998). Recently, in one report, it was shown that GmMaT2 is involved in soybean nodulation by inducing isoflavone malonylation and affecting malonyl isoflavone secretion (Ahmad et al., 2021). The ABC transporter mutant *abcg30* showed altered phenylpropanoid exudates, although it is unknown if this transporter actually transports the altered phenolics directly (Badri et al., 2009). Simultaneously, most of the transporters are involved in flavonoid exudation into the rhizosphere. The soybean plant is the most suitable model plant to study the secretion system of the flavonoid because of its large leaves and roots. Both in hydroponics and in the field, the secretion of isoflavones from soybean roots has been studied. Under hydroponic conditions, the secretion of isoflavones such as daidzein and genistein increased around 10-fold in response to a nitrogen deficit (Sugiyama et al., 2016).

Daidzein is the most dominant type of isoflavone, which is secreted throughout the lifecycle of the plant in higher amounts, from the vegetative to the reproductive phase. Whereas, in daytime conditions, the amount of daidzein and genistein in root exudates is constant. The expression of genes for transcription factors and isoflavone metabolism, however, exhibits diurnal modulation with greater daytime expression. From sunrise to noon, the isoflavone biosynthetic genes are induced by GmMYB176, a transcription factor of isoflavone biosynthesis that is strongly expressed in roots, and in the afternoon, there is a modest increase in daidzeinaglycone in the roots, as reported by Matsuda et al. (2020).

Recently, Toyofuku et al. (2021) reported that in soybeans grown under field conditions, the secretion amount of daidzein is also higher in the early vegetative stages than in the reproductive phases, but the amount is raised up to 10,000 times in comparison to hydroponic circumstances. Plant roots are capable of releasing various types of flavonoid compounds into the rhizosphere. Up until now, many studies have been conducted to determine the types and concentrations of flavonoids in root exudates. The exudates from roots contain both the aglycones and glycosides of flavonoids (Cesco et al., 2010). Plant species and cultivars, plant growth conditions, sampling methods, and nutrient supply are all important factors in the types and amounts of flavonoids (Cesco et al., 2010). Until now, little is known about the amounts of flavonoid that are actually released in the soil. Knowledge obtained from most studies conducted in hydroponic systems, sand, and water-agar supports the proposal that the amounts of flavonoids in the rhizospheric soil are minimal compared to those released by plants under *in vitro* conditions. It is concluded that experimental conditions significantly affect the active time of flavonoids in the rhizosphere. The measurement of active time and actual concentrations of specific flavonoids in the rhizosphere is problematic because of some factors like soil heterogeneity, chemical structure, and the reactivity of flavonoid molecules (Alford et al., 2007; Barto and Cipollini, 2009).

Kidd et al. (2001) conducted an experiment to quantify the amount of quercetin secreted from the roots of three maize cultivars in response to aluminium stress. Results revealed that plants pre-treated with silicon under aluminium stress significantly increased phenolic compounds, whereas only a small quantity of quercetin was measured in control plants. In a recent study, Leoni et al. (2021) looked into changes in flavonoid concentrations in plant tissues and root exudates from four legume crops that were co-cultivated with wheat crops. Results showed that both legume biomass and root exudates had a significant increment in the number of flavonoids. Daidzein, genistein, medicarpin, and formononetin concentrations changed, and these flavonoids have been reported to play a role in legume nodulation (Leoni et al., 2021). With the help of the HPLC-MS/MS method, out of 27 flavonoids, 14 have been found in lupin roots at concentrations of 0.05-736 μg/g DW, with genistein, genistin, and luteone being the most dominant (Andersen et al., 2022).

## 4 Flavonoid: a molecular planner of various associations

### 4.1 Arbuscular mycorrhiza

Phosphorus and nitrogen are two of the most important macronutrients utilized by plant for their metabolism (Singla and Garg, 2017). However, the irony is that these two elements are often found in very low quantities within the rhizosphere and act as limiting factors for growth of plants, a condition which must have been endured by early land plants shortly after their occurrence (Nascimento and Tattini, 2022). The modus operandi for minimizing the nitrogen deficiency is through symbiotic association between *Rhizobium* and legumes roots facilitated by root exudates containing primarily flavonoids.

**Figure 5:**
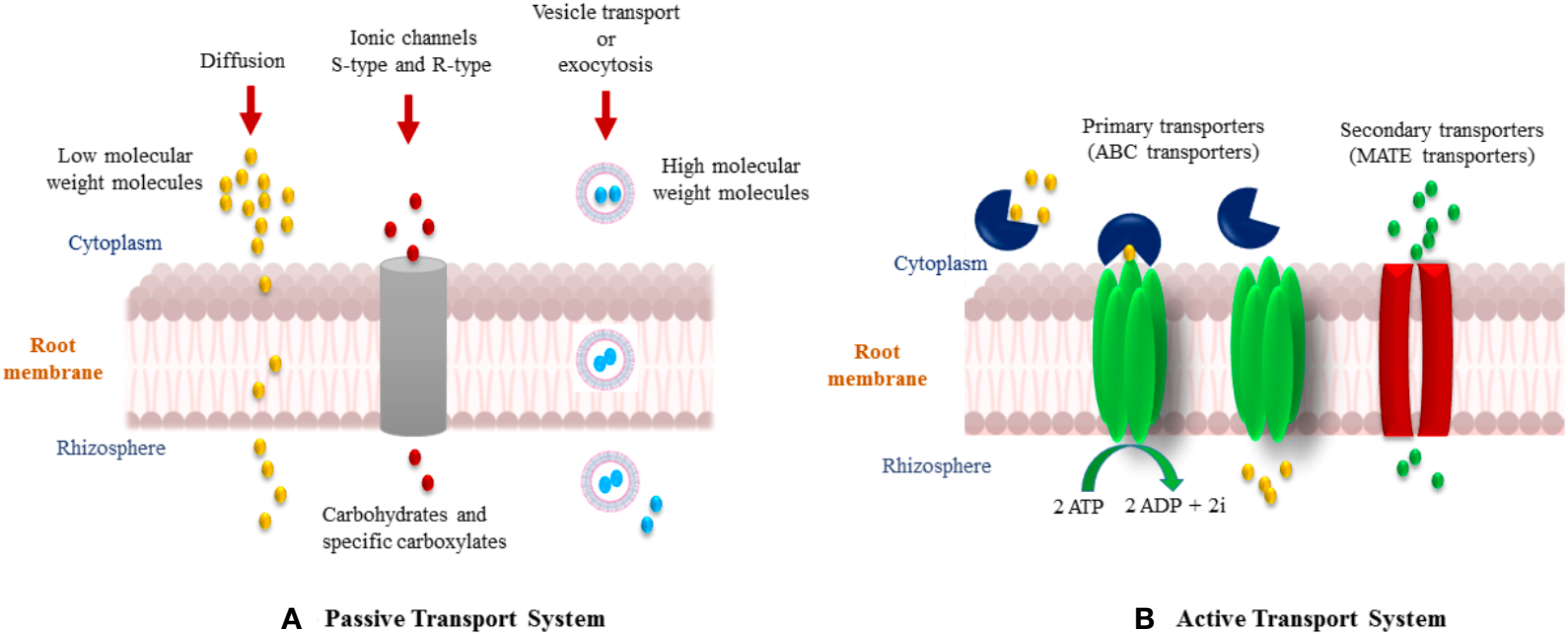
Various mechanisms of flavonoid secretion from roots into the rhizosphere. **(A)** Passive transport system, and **(B)** Active transport system.

However, phosphorus acquisition is mainly guided by another symbiotic association known as arbuscular mycorrhiza (AM). AM, consisting of fungi from zygomycetes in association with plant roots, is a highly complex network of fungal hyphae on roots acting as their extensions to host plants (Singla and Garg, 2017). This acts as a bridge between phosphorus deficient zone and phosphorus rich zone, therefore, increasing the range of plants’ roots for nutrient uptake. It has been found that flavonoids also play a crucial role in signaling between the infecting fungi and host cells for successful infection as shown in **Figure 5**. However, it has to be noted that as per some researchers, flavonoids are not necessary to form this symbiotic association as demonstrated in carrot roots, which lack chalcone synthase (Shah and Smith, 2020). Therefore, the role of flavonoids in establishing mycorrhizal association is still under scrutiny. The role of flavonoids can be seen in the initial chemical dialogue between the germinating spore with the germ tube and the host plant, releasing root exudates. The ‘branching factor’ released by the roots causes a shift in the morphology of the germ tube to enhance branching and movement toward the host plant (Singla and Garg, 2017).

A variety of flavonoids have been identified in the branching factor mainly consisting of flavonols, isoflavones, etc. It was reported by Salloum et al. (2018) that flavonoids in root exudates showed unusual trends since the fungus *Gigaspora margarita* was stimulated to form AM in carrot seedlings whereas inhibited the same in pea for the same fungus. However, various studies report that the hyphal branching is inhibited or stimulated not only on the basis of host-microbe specificity but also on the concentration of flavonoid secreted since the same stimulatory flavonoids became inhibitory in larger concentration (Singla and Garg, 2017). *In-vitro* studies on AM fungal association between *Arbutusunedo* and *Tuberborchii* by Gomes et al. (2021) found that quercetin concentrations up to 2 μM were found to be optimum for successful colonization and concentrations above 7 μM of the same compound had inhibitory effects though quercetin is considered to be a widespread flavonoid in mycorrhiza forming plants.

Flavonoids can be an inducer or non-inducer depending upon whether the germ tube coming in contact with the exudate compounds is compatible with the host or not. A variety of flavonoids such as hesperetin, naringenin, and formononetin have been found to serve as signals for initiating mycorrhizal association with the effect of formononetin reportedly enhancing the dry weight, tuber production, and efficiency of nutrients (Bag et al., 2022). Tian et al. (2021) found that flavonoids such as quercetin and quercitrin were found to be in large quantities and played a pivotal role in enhancing mycorrhizal association in introduced *Triadica sebifera* population in comparison to the native population, making the root exudates of the former more potent than the latter in inducing symbiosis. Under favorable conditions during the pre-symbiotic phase, the germ tube shows multiple branching and a directed growth towards the root system once they come in contact with ‘branching factor’ flavonoids. This leads to a release of *Myc* factor by fungal hyphae helping with AM establishment by grading down the defense mechanisms (Singla and Garg, 2017).

Auxins are also known to play a pivotal role branching of hyphae and arbuscule establishment. Since flavonoids are one of the key modulators of auxin hormone, Nascimento and Tattini (2022) have argued that flavonoids play a major role in regulating the transport of auxins to colonized cells, though the molecular aspects of this endogenous regulation need to be explored. After the infection, characteristic structures known as arbuscules are formed within the cortical cells for the exchange of metabolites. It has been found that certain flavonoids such as naringenin in the case of beans helped in increasing the number of vesicles and arbuscules *per* cell (Singla and Garg, 2017). It should be noted that the function of flavonoids in promoting association between symbionts also comes with an initial defense response from the host by the production of certain defensive flavonoids such as phytoalexins (medicarpin). After some time, the host becomes aware of the agent’s “intentions” and results in lowering the concentration of defensive compounds. Thus, it can be concluded that flavonoids act as molecular messengers between the fungal component and host in mycorrhizal association not only during the pre-symbiotic phase but also during intracellular differentiation of haustorial structures of hyphae, aiding in cell-to-cell interaction. Studies performed on improved (I-1) and unimproved genotypes of soybean (UI-4) suggest that exogenous application of flavonoids improved arbuscular formation and fungal colonization (Salloum et al., 2018).

### 4.2 Non-rhizobial bacterial association mediated by flavonoids

The microbial population present within the rhizosphere is subjected to various chemicals produced by the plants which further determine the distribution of population of these microbes. The phenomenon of bacterial colonization can be considered as an amalgamation of soil health and chemical composition of root exudates, which is genetically determined (Chandran et al., 2021). These molecules, sometimes being similar to microbial signaling molecules (Abedini et al., 2021), can not only establish a mutualistic relationship between the two parties involved, but can be an ‘SOS’ call to recruit certain bacterial population when under chemical and biological crisis such as metal toxicity or pathogen attack. Therefore, it can be said that the change in the root exudates when subjected to a particular stress can help in changing the dynamics of the microflora of rhizosphere according to the need of the plant host with respect to its environment (Abedini et al., 2021; Chandran et al., 2021). Rolfe et al. (2019) advocated a ‘cry for help’ hypothesis which states that roots of the plants are exposed to a variety of pathogenic and non-pathogenic microbes. On encountering stresses, biotic and potentially abiotic, roots of the plant produce exudates consisting of microbe-specific compounds which help in immediate recruitment of beneficial microbes from the soil microbiome to cope with the stress. Numerous findings by researchers suggest that such activities are not only confined to the immediate plant under distress but the benefits also extend to the successive generations (Rolfe et al., 2019). There are certain differences between roots and shoots when taking immunological responses into consideration. This has been speculated to be due to high exposure of roots to microbiome in comparison to shoots and the lack of chloroplast in roots which does not allow them to produce protective free radicals of oxygen and nitrogen (Rolfe et al., 2019).

The ‘immune’ responses of roots help in the identification of type of microbe under the radar and help in the deployment of specific secondary metabolites to suppress the ill effects caused due to its infection (Rolfe et al., 2019; Meena et al., 2023a; Meena et al., 2023b; Zehra et al., 2023). However, more elaborate studies are to be conducted to know the extent of validity of the hypothesis since pieces of evidence on abiotic stress tolerance regarding the aforementioned hypothesis are less (He et al., 2018). Flavonoids have been found to be a fascinating member of these chemical signals with their role in nitrogen fixation extensively studied in leguminous plants. It has been surprisingly found that there are molecules produced by plants, which are not involved in root nodulation process but are classified under flavonoids and help in survival of plants under biotic and abiotic stresses. Under the presence of a potential harmful microbe, phytoalexins are known to be produced within the plant and are supposedly stored in inactive state as an instant remedy to future infections (Bag et al., 2022). *Pseudomonas* sp. inhibit the further division of pathogen by producing phytolexins (coumestrol) in bean plants (Shah and Smith, 2020). Though, it has been found that certain microbes have learnt to bypass this defense mechanism by adapting to the specific flavonoids (Bag et al., 2022). Even though, flavonoids have been known to be stress mediated compounds which further help in recruiting microbial allies.

He et al. (2022) reviewed that the concentration of flavonoids in root exudates cannot be always determined in accordance with ‘Cry for help’ hypothesis, especially for abiotic factors. Studies conducted upon *Arabidopsis* plant to check for the effect of flavonoids in helping the plant survive dehydration stress suggest that the recruitment of *Aeromonas* sp. H1 strain under dehydration stress was decreased, if not, similar for the test and control plants though the concentration of flavonoids increased under stress suggesting that it is not a distress call as hypothesized (He et al., 2022). It was found that flavonoids, specifically naringenin, played a pivotal role in attracting the *Aeromonas* H1 strain by increasing its flagellar motility. Ma et al. (2014) revealed that the concentration of flavonoids, schaftoside in particular, increases during a period of drought. This was found to be possible due to the special structure of flavonoid which helps not only in countering the production of ROS but also consuming if formed, thus protecting from oxidative stress. Nakabayashi et al. (2014) also observed similar type of results in the case of *Arabidopsis* under drought stress. The studies on the process of nitrogen fixation have been done mainly on the associations between nitrogen-fixing microbes and leguminous crops, the latter regarded as the ones making nitrogen available to the soil.

However, *in-vitro* studies on rice plants by Shamala Tirimanne et al. (2018) suggest that the application of biofilm made of *Azorhizobium caulinodans* and *Aspergillus* sp. along with naringenin in a concentration of 10^-5^ M or 10^-4^ M to the roots of rice enhanced the major yield parameters of rice. Thus, it can be concluded that naringenin is an important *nod* gene inducer, and the formation of associations between diazotrophs and non-leguminous plants can play a pivotal role in diversifying the process of nitrogen fixation. Yu et al. (2021) suggests through their experiment on maize that the root exudates produce metabolites consisting of flavonoids, specifically flavones, which help in attracting microbes of Oxalobacteraceae. This association assists the maize plant under nitrogen-deficient condition and also promotes the formation of lateral roots. QS molecules produced by bacteria can also be detected by plants and secrete metabolites which can repel or attract microbes. The position of flavonoids, as discussed above, has been ambiguous with respect to whether they are inhibitors or promoters of microbial association but they do influence the rhizobial microbiome (Bag et al., 2022).

### 4.3 Bioavailability and persistence of flavonoids in rhizosphere

**Figure 6:**
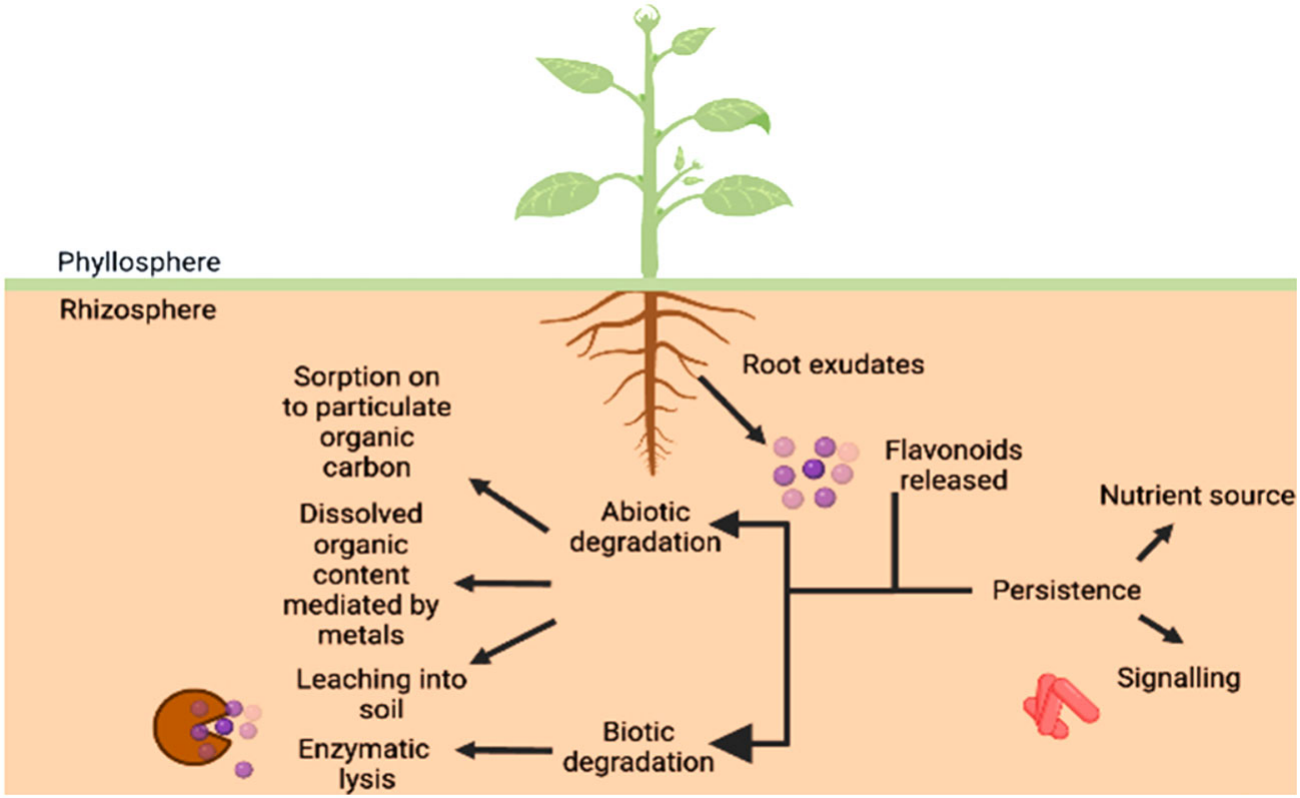
Bioavailability of flavonoids in the rhizosphere. The persistence of flavonoids provides the microorganisms a nutrient source as well as a signalling mediator, therefore, effective communication taking place between host and microbe. The non-availability of the same can be attributed to sorption and desorption to particulate organic content, leaching and dissolved organic content, comprising of abiotic factors and enzymatic lysis being the biological factor.

A number of research studies have unequivocally proved that secondary metabolites such as flavonoids play a key role in mediating the communication between plants and beneficial microbes. However, various biotic and abiotic factors affecting the parameters of soil such as mineral concentration, since calcium is seen to protect the flavonoids from degrading (Sugiyama and Yazaki, 2014) and govern the duration of this signaling event and even the persistence of flavonoids in the rhizosphere which is summarized in **Figure 6**. The flavonoids exist in a conjugated form with carbohydrate moiety known as glycoside in the roots. As they are released from the roots into the soil, the glycosidases of microbes attack these flavonoid-conjugates producing free flavonoids (**Figure 6**). However, the concentrations of both free and bound phenolics have been reported in the soil. The biotic degradation has been reported to be carried out by microbes such as *Pseudomonas putida* and various *Rhizobium* species which involves migration of B ring from 3^rd^ position to 2^nd^ position on C ring (in case of isoflavonoids), breakdown of C ring, formation of a temporary chalcone molecule and the result being A and B ring hydroxylated aromatics (Shaw and Hooker, 2008). While the contribution of biotic factors has been studied in detail with respect to inhibition of the molecular communication between the two entities, the abiotic regulators have remained unexplored. The root exudates released are subjected to various processes such as scavenging by soil microbes, polymerization, transformation into less toxic compounds, etc. Generally, flavonoids are subjected to enzymatic degradation by the microorganisms present in the soil since these compounds are a source of nutrients for them. Therefore, some forms of the metabolite are present for longer durations while others are eaten up within a matter of hours.

Since the microbial populations vary as *per* the seasons, it is also fit to say that the flavonoid degradation can be regarded as a function of seasons as well as reported by Sosa et al. (2010) where longer durations were reported during autumn season for exudates from *Cistus ladanifer*. The results obtained by Sosa et al. (2010) provide an insight to a higher persistence of apigenins and other flavonoids secreted by *C. ladanifer* in the soil. It was concluded that the compounds were trapped within the organic matter, one of the abiotic factors unexplored, present in the soil helping them reside in rhizosphere for a long time. It was realized by Sugiyama and Yazaki (2014) that the addition of manure to the soil, lead to an increase in the flavonoid released by roots, though no major difference was observed but studies done by Del Valle et al. (2020) found that the content of organic carbon present in the soil plays a major role in bioavailability of flavonoids to the parties involved in dialogue.

Their findings suggest that organic carbon (OC), more specifically dissolved OC, influences the signal availability by reacting with the signalling molecule involved in the presence of a metal mediator such as Mn^3+^ and decreases the efficacy of the compound. This was speculated to be an adaptation to conserve energy during nutrient abundance in soil instead of forming unnecessary associations. Flavonoid glucosides were reported to be more readily degraded than other forms of flavonoids (Sugiyama and Yazaki, 2014) owing to their rapid enzymatic hydrolysis. The different structures of flavonoids such as in case of formononetin and naringenin can result in differential reactions with organic matter as reported by Del Valle et al. (2020). Therefore, the structure and chemical makeup of flavonoids play a pivotal role in determining their persistence in soil.

### 4.4 Flavonoids-mineral acquisition role

Nitrogen and phosphorus are notorious in terms of their bioavailability within the rhizosphere. With a high immobilization/mineralization ratio, these minerals are generally deficient in the soils of crop land. The uptake of nitrogen by the formation of symbiotic association between *Rhizobium* and leguminous crops is one of the best studied mechanisms. Certain compounds under flavonoids (isoflavonoids) act as chemical messengers secreted by the roots and seeds of plants such as *Phaseolus vulgaris*, *Glycine max* which attract the *Rhizobia* and stimulate bacterial *nod* genes inducing root nodule formation (Cesco et al., 2010). Furthermore, flavonoids have a polar role in acquisition of important minerals such as iron (Fe), phosphorus (P) and certain micronutrients.

Phosphorus is an important macronutrient utilized in plants as a component of nucleic acids, ATP and phospholipids. It is not easily available in soil due to its immobilization by precipitation and association with organic compound. Therefore, it requires means such as enzymatic degradation (Cesco et al., 2010). Plants need to develop various mechanisms to acquire Pi from P-sorbing soil (Xiong et al., 2022). The phosphorus deficiency is dealt by plants through the release of a particular class of compounds such as flavonoids. Apart from the promotion of mycorrhiza formation, there is another method of maintaining supply of P to plants. Usually, certain chelating carboxylates are produced by the roots under phosphorus deficiency which binds to phosphorus and increase their mobilization (Ding et al., 2021). Certain flavionoids are also proposed to function as chelating agents (Cesco et al., 2010). They mobilize the phosphorus by splitting the P bound to metals such as Fe and Al (Ding et al., 2021) and forming complexes with the metals therefore making the bound P available (Cesco et al., 2012). The increased production of naringenin, quercetin and genistein by *Lupinus albus* from the cluster roots formed during P-deficiency and in the roots of alfalfa (Cesco et al., 2012; Xiong et al., 2022) are well observed phenomena supporting this mechanism since a large amount of metal chelators are required for mobilization of phosphorus reserve. Another mechanism involves protection of the metal chelating carboxylates from microbes as seen in the case of *Lupinus albus* where the increase in citrate concentration is coupled with isoflavonoid secretion inducing microbial respiration (Cesco et al., 2010).

Iron (Fe) is an essential nutrient, which plays a major role in light reactions of photosynthesis. Iron in soil can occur as in the following forms, Fe (II) state in primary minerals, Fe (III) state found in crystalline state (Cesco et al., 2010; Colombo et al., 2014). The stability of crystalline states such as hematite is high therefore very less mobilization is seen. However, the bioavailability of Fe in soil is dependent upon soil pH. Iron is usually present in Fe (III) form but it forms highly stable complexes such as hydroxides and oxides therefore rendering the nutrient unavailable (Chai and Schachtman, 2022). It has been seen that the acidic or reducing conditions makes Fe more available in soil (Colombo et al., 2014) by converting Fe (III) to Fe (II) which is a more available form (Chai and Schachtman, 2022). Furthermore, iron also binds to organic compounds and exists as a complex (Colombo et al., 2014). Since, iron has physiological importance in plants; its availability plays a crucial role in the optimum growth and development of crop. The mechanism of uptake of Iron can be divided into two forms depending upon the type of crop *i.e.*, Dicots including non-graminaceous monocots constituting the first group and monocots the second group (Colombo et al., 2014). The strategy of the former group involves primarily the reduction of Fe (III) to Fe (II) (Chai and Schachtman, 2022) which involves various methods such as efflux of protons and organic compounds, reduction of Fe (III) by a plasma membrane bound NADPH dependent ferric chelate reductase (Cesco et al., 2010). The strategy of the latter group *i.e.*, Poaceae involves synthesis of compounds known as phytosiderophores which bind to Fe (III) and form complex which is taken up by plants by transporters. As per Chai and Schachtman (2022), rice has been the only candidate known to take up Fe (II) while following the chelation strategy.

As discussed above, a common trend of reduction and chelation can be observed and it can be rightly concluded that reducing and chelating conditions would lead to more mobilization of Iron. Flavonoids have both of these characteristics inclusive to their structure, as seen in case of quercetin, genistein that can reduce Fe (III) *in vitro* (Cesco et al., 2010). They also have an ability to chelate with Fe to form complexes as reported by Ferlazzo et al. (2016) in their study which emphasized on the anticancer properties of flavonoid rich citrus fruits owing to their chelation to Fe (III) forming complexes. It was also seen by Zamboni et al. (2012) that one of the responses of tomato plants to iron deficiency was the upregulation of *flavonoid-3-hydroxylase* gene. Therefore, these findings support the proposed additional role of flavonoids in having an important function in mediating the bioavailability of Iron in soil but as concluded by Merry et al. (2022), extensive study is required to finalize its modus operandi.

### 4.5 Flavonoids as alleviating candidates against biotic stress

Plants are sessile organisms lacking the ability to hide from an attacker or flee from one and are constantly in danger from possible infections just like all other multicellular species. In nature, plants are constantly exposed to an array of challenges incited by pathogenic microbes such as fungi, bacteria, viruses, and nematodes. To stave off such microbes, plants have a well-furnished, intricate series of defense mechanisms. Plants overcome these stresses through the production of pathogenesis-related (PR) proteins, activation of defense-related genes, the formation of reactive oxygen species (ROS), cell-wall reinforcement, and the synthesis and accumulation of antimicrobial compounds (Houston et al., 2016; Meena et al., 2022; Mittler et al., 2022). Plant species secrete an array of chemical substances when they are exposed to pathogenic microbes. These chemical substances, which are low-molecular-weight secondary metabolites with antimicrobial properties that are produced and accumulated in plants after exposure to stresses, are commonly known as phytoalexins (Bizuneh, 2021; Singh et al., 2023). Phytoalexins are a group of potential compounds that have biocontrol activity against a number of plant pathogens and are considered molecular markers of disease resistance (Jeandet et al., 2014; Meena et al., 2020; Sharma et al., 2022). Different chemical compound classes, such as terpenoids, phenolics, stilbenoids, alkaloids, flavonoids, and many others, are described as potential groups of phytoalexins (Chripkova et al., 2016; Meena et al., 2017; Yang et al., 2018).

Flavonoids are well-known polyphenolic secondary metabolite compounds among various phytoalexins, which have a pivotal role in the defense system. They are mainly species-specific secondary metabolic compounds (Taulavuori et al., 2016), and their biosynthesis is mostly dependent on certain factors like crop growth stage, plant species, and type of stress (Schijlen et al., 2004; Li et al., 2020). Flavonoids have a variety of antifungal, antibacterial, antiviral, and other potential activities as discussed below in the subsections.

### 4.6 Flavonoids as antifungal, nematicides and antibacterial agents

Plant pathogenic fungi are a real scourge to agricultural crop production because they cause different types of diseases in crop plants. To combat the harmful effects of fungal pathogens, plants have the capacity to produce an array of intricate defense mechanisms, including the formation, accumulation, and secretion of phytoalexins, especially flavonoids. Grayer and Harborne (1994) conducted a survey to compile a list of phenolic chemicals from higher plants that have antifungal properties. He discovered that flavonoids are the most common class of phenolic chemicals involved. Skadhauge et al. (1997) reported that the two such members of this group, proanthocyanidins and dihydroquercetin, in barley mutants show resistance against *Fusarium* species. There are three mechanisms that contribute to resistance to pathogen infection. The first is microbial enzyme crosslinking; the second is inhibition of microbial enzymes (cellulases, xylanases, and pectinases); and the third is the formation of a hard-crystalline structure against pathogen attack. Isoflavones, flavonols, and flavanones have been reported for their role in suppressing spore germination and mycelial growth of fungal pathogens (Morkunas et al., 2011; Ramaroson et al., 2022). Increased accumulation of isoflavonoid phytoalexins directly contributes to brown disease resistance in alfalfa (He and Dixon, 2000). Genistein, a yellow lupine isoflavone, was found to be an effective phytoalexin in reducing the severity of *F. oxysporum* disease (Morkunas et al., 2005).

In rice, biosynthesis of sakuranetin (a flavanone) was shown to enhance disease resistance to three major rice pathogens: blast incited by *Magnaporthe grisea* (Hasegawa et al., 2014), sheath blight incited by *Rhizoctonia solani* (Katsumata et al., 2018), and bakanae incited by *Fusarium fujikuroi* (Siciliano et al., 2015). Citrus flavonoids hesperetin and naringenin provide induced resistance against *Phytophthora citrophthora*-caused citrus brown rot (Del Río et al., 2004). The sorghum plant is a rich source of antifungal flavonoids; among them, 3-deoxyanthocyanidins belong to a unique class of flavonoid phytoalexins that play an essential role in disease resistance (Ibraheem et al., 2010; Tugizimana et al., 2018; Förster et al., 2022). The 3-deoxyanthocyanidins and luteolin (a flavone) in sorghum were shown to inhibit the spore germination of anthracnose disease (Du et al., 2010). Wang et al. (2020) reported that flavonols and anthocyanidins were found effective in increasing plant resistance against anthracnose disease in transgenic sorghum lines (*SbF3H1*).

Parasitic nematodes infect the cysts or galls on roots which further reduces the crop yield (Mandal et al., 2021). The invasion of nematodes in plant root system induces the secretion of flavonoids. Coumestrol (phytoalexin), glyceollin (isoflavonoid) as nematicides is secreted by root knot of the plants against *Pratylenchus penetrans*, *Meloidogyne incognita*, respectively (San Chin, 2019). In oat *O*-methyl-apigenin-*C*-deoxyhexoside-*O*-hexoside (a flavone-*C*-glycoside) secreted as major nematicides (Shah and Smith, 2020). Till now, the defence mechanism of plant root against nematodes by flavonoids is unclear. Some flavonoids like Coumestrol, daidzein, phaseollinisoflavan and kievitone were identified from plants against certain pathogenic bacteria such as *Pseudomonas mars-prunorum*, *Pseudomonas* phaseolicola, *Pseudomonas glycinea*, *Pseudomonas lachrymans*, *Xanthomonas* and *Achromobacter*. Phaseollinisoflavan and kievitone secreted by plants against *Xanthomonas* and *Achromobacter* species (Wyman, 1978). Root of *Erythrina poeppigiana*, secret isoflavonoid against *Staphylococcus aureus*.

## 5 Conclusion and future prospects

The soil microbiome is essential for increasing the fitness of the plants. The rhizosphere consists of both pathogenic and non-pathogenic microbes, the former being utilized by the plants when under distress such as drought, pathogen attack and/or herbivory. The chemical dialogue between the microbial community and the plant host is governed by secretions known as root exudates which consist of primary and secondary metabolites. This serves as a carbon source to the microbiome and helps in attracting microbes to the roots and flavonoids is an important group of compounds present as its constituents. The flavonoids are secreted majorly in the form of flavonoid glycosides though aglycones are known for their role in root nodulation. The glycosylation enhances the mobility and distribution whereas the methylation of the same facilitates its entry into cell. The secretion of this class of compounds into the rhizosphere is done by ABC transporters which is the basis of active release and passively they are released *via* root injury or any other harm to the root tissues.

In the spirit of novelty, in this review, the authors explained the indispensable role of flavonoids at an advanced level in secretion mechanisms, including active and passive mechanisms, into the rhizosphere. More specifically, this review shows strong evidence of flavonoids roles in plants against pathogens and their pivotal contribution to biotic stress resistance. Moreover, we systematically summarized the flavonoids roles at the next level in nutrient acquisition, bioavailability, and persistence in the rhizosphere. Still, however, very little information is available about the role of flavonoids in root nodule formation, which we discussed in detail based on the available literature until now. This review would surely facilitate a profound perception of flavonoids for the scientific community or those who work on flavonoids. After gaining better knowledge of flavonoids roles and mechanisms, it will be easy to target the next generation of flavonoids, which may have a multifarious approach.

## Author contributions

GAK: Conceptualization, Methodology, Writing – original draft, Writing – review & editing. SK: Methodology, Writing – original draft, Writing – review & editing. RB: Conceptualization, Methodology, Writing – original draft, Writing – review & editing. MM: Conceptualization, Data curation, Formal analysis, Investigation, Methodology, Software, Supervision, Validation, Visualization, Writing – original draft, Writing – review & editing. PS: Conceptualization, Data curation, Investigation, Methodology, Software, Supervision, Validation, Visualization, Writing – original draft, Writing – review & editing. CSS: Writing – original draft, Writing – review & editing. AY: Validation, Writing – review & editing.
